# Rational engineering of an antifreeze-based biomaterial with dual-enhanced ice-binding and delivery for high-efficiency cryopreservation

**DOI:** 10.1016/j.jare.2025.12.008

**Published:** 2025-12-08

**Authors:** Yishan Fu, Yangyang Li, Fei Pan, Sahibzada Muhammad Aqeel, Song Liu, Jian Chen

**Affiliations:** a Science Center for Future Foods, Jiangnan University, Wuxi 214122, China; b School of Biotechnology, Jiangnan University, Wuxi 214122, China; c State Key Laboratory of Resource Insects, Institute of Apicultural Research, Chinese Academy of Agricultural Sciences, Beijing 100093, China; d JiaXing Institute of Future Food, Jiaxing, Zhejiang 314000, China

**Keywords:** Snow flea antifreeze protein, Cryopreservation, Structural optimization, Transmembrane delivery, Universality

## Abstract

**Introduction:**

Reliable cell cryopreservation is essential for biomedical research and cell-based therapies. Although dimethyl sulfoxide (DMSO) is widely used as a cryoprotectant, its cytotoxicity and clinical incompatibility limit its application, creating an urgent need for safer alternatives. Antifreeze proteins (AFPs) offer favorable biocompatibility and reduced cytotoxicity, making them promising candidates for next-generation cryoprotectants.

**Objectives:**

This study aims to develop a high-performance cryoprotective biomaterial via rational engineering of snow flea AFP (SfAFP), establish an effective DMSO-free cryopreservation strategy, and verify its applicability across multiple cell lines.

**Methods:**

rSfAFP-MU was generated by site-directed mutagenesis (T16G, A19G, S22G) of SfAFP. Comprehensive physicochemical (thermal hysteresis, particle size, hydrophobicity, morphology) and functional (EA.hy926 post-thaw recovery) characterizations were performed. Molecular dynamics simulations identified key ice-binding interfaces, guiding alanine substitutions at D75, P78, P81 to test geometry. To enhance intracellular delivery, the TAT peptide was fused to rSfAFP-MU’s N-terminus via a (G_4_S)_3_ linker (yielding rTAT-GS-MU), whose localization was confirmed by confocal microscopy. Cryopreservation efficacy was tested in EA.hy926, A549, and HEK293 cells.

**Results:**

The rSfAFP-MU mutant demonstrated significantly enhanced thermal hysteresis (5 °C vs. 2.19 °C for wild-type SfAFP) and increased EA.hy926 post-thaw recovery by 10.20 % compared to SfAFP. Molecular dynamics simulations revealed that enhanced ice-binding affinity stems from optimized hydrophobic surface intersection angles between monomers, a finding validated by activity reductions in targeted mutants. Confocal imaging confirmed efficient intracellular uptake of rTAT-GS-MU, which correlated with improved cryopreservation efficacy. rTAT-GS-MU achieved 74.77 % EA.hy926 post-thaw recovery (comparable to DMSO) with improved mitochondrial and nuclear DNA integrity and demonstrated efficacy in A549 and HEK293 cells.

**Conclusion:**

Through rational design, we converted a natural AFP into a high-performance bioactive material that enables efficient cryopreservation of multiple cell lines. This DMSO-free strategy addresses key limitations of traditional methods, offering a valuable alternative for biomedical use.

## Highlights

• Engineered SfAFP restores the G-G-X motif, boosting TH and EA.hy926 post-thaw recovery.
• MD simulations and mutagenesis reveal optimized hydrophobic ice-binding geometry.
• rTAT-GS-MU enables intracellular delivery, achieving 74.77% EA.hy926 post-thaw recovery.
• Validated across EA.hy926, A549, HEK293 cells, demonstrating broad applicability.
• DMSO-free cryopreservation delivers comparable recovery with reduced mito/DNA damage.

## Graphical abstract

Schematic illustration of the rational design, cryoprotective mechanism, and broad applicability of an engineered snow flea antifreeze protein (SfAFP). Site-directed mutagenesis (T16G/A19G/S22G) produced the high-activity mutant rSfAFP-MU; MD simulations guided dimeric ice‑binding interface optimization, expanding the hydrophobic ice‑binding surface and strengthening ice affinity. N-terminal fusion with a TAT cell-penetrating peptide via a (G_4_S)_3_ linker yielded rTAT-GS-MU, enabling efficient intracellular uptake and binding of nascent intracellular ice crystals to suppress growth and preserve mitochondrial and nuclear integrity during freezing. In DMSO-free cryopreservation, rTAT-GS-MU achieved post-thaw recovery comparable to DMSO across EA.hy926, A549, and HEK293 cells, indicating broad potential for cell therapy and biobanking.

**Figure:**
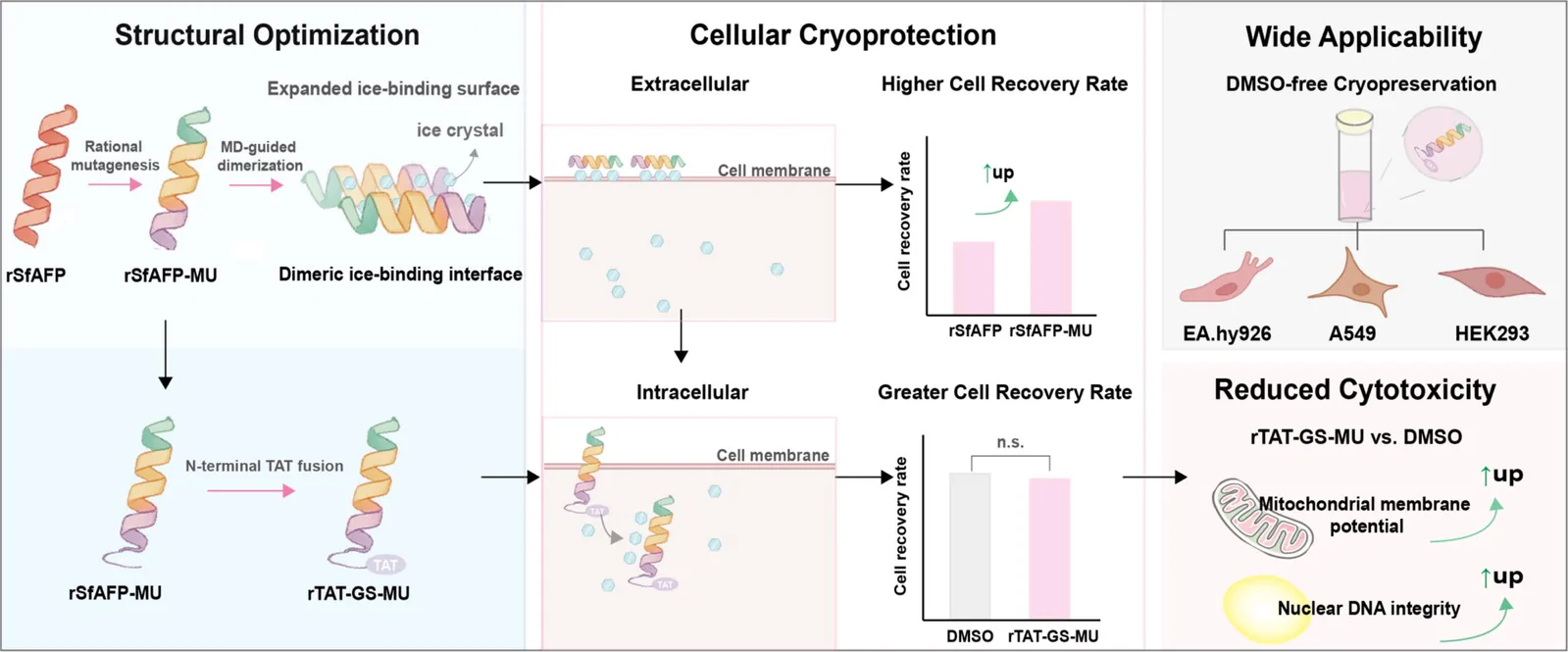

## Introduction

Long-term storage of viable cells underpins a broad spectrum of biomedical applications—from drug screening and precision oncology to regenerative medicine and allogeneic transplantation [1]. Cryopreservation, a technology that suspends cellular metabolism indefinitely while preserving genomic and functional integrity [2], relies critically on cryoprotective agents (CPAs) to suppress lethal ice nucleation and growth [3]. Without CPAs, 70–90 % of intracellular water crystallizes upon cooling, generating mechanical stress that ruptures lipid bilayers and organelle membranes, while triggering osmotic shock and apoptotic signaling cascades [4]. Dimethyl sulfoxide (DMSO) remains the gold-standard permeable CPA because it colligatively lowers the freezing point and limits ice propagation. However, DMSO’s clinical utility is compromised by dose-dependent cytotoxicity that manifests as membrane permeabilization, cytoskeletal disruption, and mitochondrial dysfunction; residual DMSO metabolites have further been implicated in epigenetic aberrations and lineage infidelity in stem-cell-derived products [5,6]. This creates an urgent demand for next-generation DMSO-free CPAs, ideally bioactive materials, that match or exceed DMSO’s cryoprotective efficacy while eliminating its toxic liabilities.

Current DMSO-free CPAs include synthetic polymers (polyvinyl alcohol, poly(2-oxazoline)) [7], two-dimensional nanomaterials (graphene oxide (GO), MXene) [8], and glycoprotein-mimetic peptides [9] or antifreeze proteins (AFPs) [10]. Among these, AFPs—evolutionarily tuned for precise ice control—are especially promising as cryoprotective biomaterials [10]. First isolated from *Antarctic Notothenioid* fish in 1969 [10], AFPs have since been identified across diverse psychrophilic taxa (insects, plants, bacteria, fungi), characterized by their ability to regulate ice crystal growth via an adsorption–inhibition mechanism: they bind specific ice planes through lattice-matching motifs, generating local curvature that thermodynamically impedes further ice propagation [11]. Unlike small-molecule cryoprotectants (CPAs), their ice-interactive action is largely extrinsic to cellular components, resulting in minimal cytotoxicity—a key advantage for biological applications. For example, AFPs from *Anatolica polita* beetles and *Glaciozyma antarctica* yeast have improved post-thaw recoveries of bacteria, fibroblasts, and primary mammalian cells [12]. However, most AFPs suffer from two critical limitations as functional biomaterials: modest thermal hysteresis (TH) activity (≤2 °C) and extracellular confinement, leaving intracellular ice formation unchecked [13]. Enhancing their intrinsic antifreeze potency and intracellular accessibility is thus essential to advance their translational potential as cryoprotective materials.

**Fig. 1:**
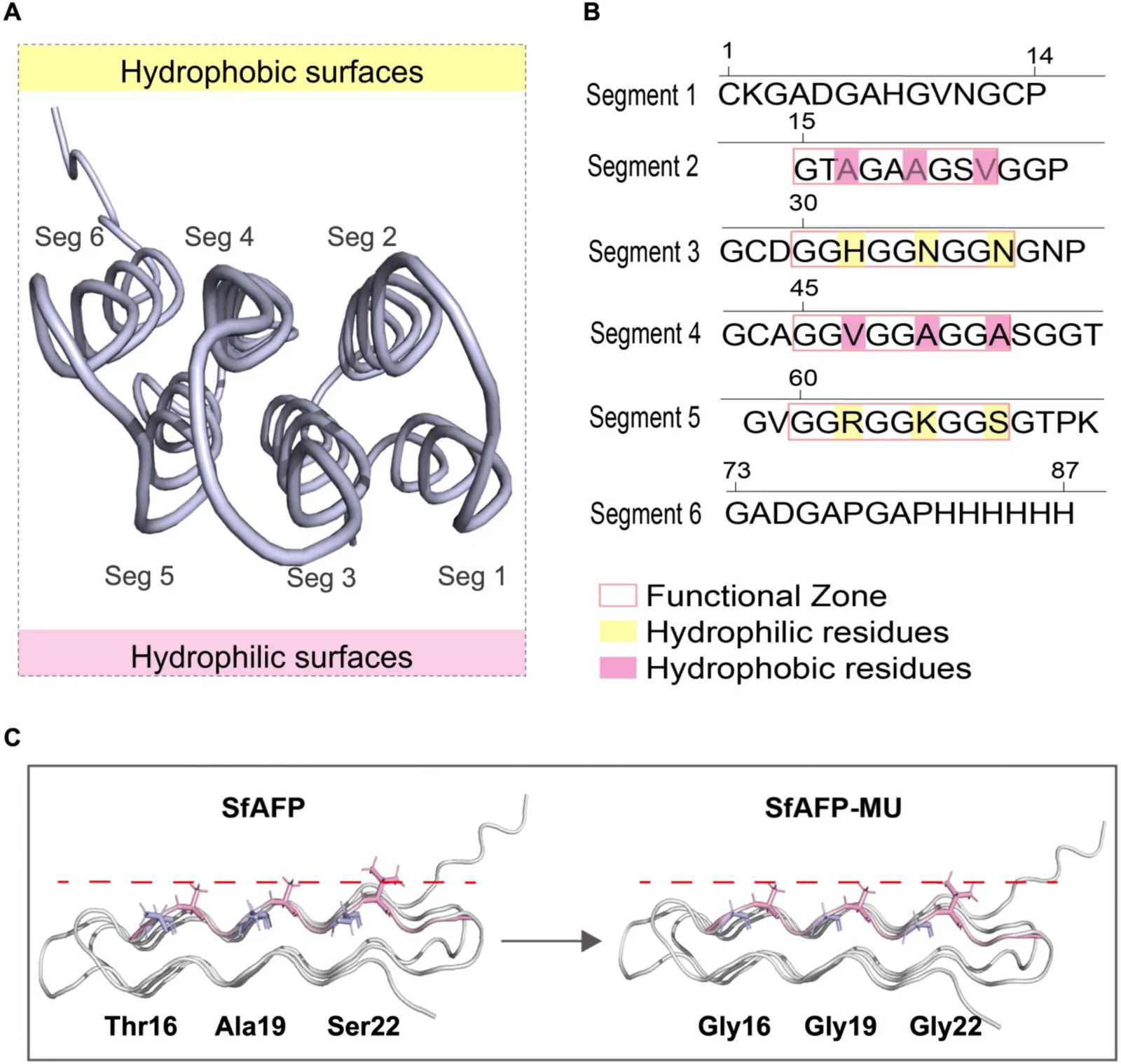
Structural characteristics and engineered optimization of SfAFP. (A) Crystal structure of SfAFP (PDB: 2PNE) showing the distribution of hydrophilic and hydrophobic surfaces to illustrate the potential ice-binding interface configuration. (B) Amino acid sequence analysis of SfAFP. The characteristic “G-G-X” repeat motifs marked in red boxes exhibit regular arrangement, forming the core functional units of the AFP. (C) Rational engineering of the Seg2 fragment: restoration of “G–G–X” periodicity (T16G, A19G, S22G mutations) significantly improved the regular arrangement of hydrophobic amino acids (pink), enhancing the structural basis for ice anchoring.

Snow flea-derived AFP (SfAFP) is a 6.5 kDa hyperactive protein that adopts a six-stranded polyproline II (PPII) helix (PDB: 2PNE) [14]. It has demonstrated that two solvent-exposed hydrophobic surfaces (segments 2 and 4) that function as ice-binding sites and two hydrophilic surfaces (segments 3 and 5) that sterically block ice propagation (Fig. 1A) [15,16]. For AFPs, repeat sequence periodicity is a key structural determinant of ice-binding efficiency—for instance, *Tenebrio molitor* AFP uses stepwise “T-X-T” threonine repeats to block water ordering, forming hydrophobic platforms and dynamic hydrogen-bond networks [17]. Comparative sequence analysis shows that in the major functional regions (seg2-seg5) of SfAFP. Segments 3–5 contain typical “G-G-X” repeats (where G represents Gly and X includes Ala, Arg, Asn, Asp, His, Lys, Ser, Thr, or Val), while seg2 features atypical G-T-A/G-A-A/G-S-V motifs (Fig. 1B) that disrupt helical periodicity and impair the ice-anchoring efficiency of hydrophobic amino acids (Fig. 1C). This structural defect in seg2 provides a rational target for engineering SfAFP as a high-performance cryoprotective biomaterial: optimizing seg2 motif periodicity to match ice lattice geometry should enhance its ice-binding and antifreeze activity.

A major limitation of most AFP-based cryoprotectants is their focus on extracellular ice regulation, leaving intracellular ice formation—a critical driver of cryodamage—largely unaddressed. To overcome this, cell-penetrating peptides (CPPs) offer a proven strategy to enhance intracellular accessibility of bioactive proteins. TAT peptide (derived from the HIV transactivator of transcription, GRKKRRQRRRPPQ) [18] and the synthetic peptide Pep-1 (KETWWETWWTEWSQPKKKRKV) [19] are two widely studied examples of CPPs. Both mediate protein delivery primarily through electrostatic interactions with the cell membrane, facilitating endocytosis. TAT is typically covalently fused to cargo proteins, enabling efficient transport of functional proteins (e.g., GFP, RNase A) [20]; Pep-1 forms non-covalent complexes via physical mixing, with demonstrated success in delivering AFPs [19] and β-galactosidase [21]. This study aims to enhance SfAFP's intracellular antifreeze efficacy by fusing or conjugating it with CPPs.

Our previous work expressed recombinant SfAFP (rSfAFP) in *Komagataella phaffii* GS115 and confirmed its protective effect on human umbilical vein fusion cells (EA.hy926), yet its antifreeze activity requires enhancement. Here, we engineered SfAFP via rational modification of conserved motifs (T16G, A19G, S22G) in the seg2 region to restore sequence periodicity—aiming to enhance ice-binding efficiency and TH activity as a functional biomaterial. Using molecular dynamics simulations and site-directed mutagenesis, we dissected the structure–activity relationship underlying its antifreeze mechanism. To further optimize cellular interaction, we conjugated CPPs for intracellular delivery. The engineered protein's cryoprotective efficacy was systematically evaluated in EA.hy926 cells (assessing recovery rate, morphology, mitochondrial membrane potential, and nuclear DNA integrity) and validated across A549 and HEK293 cells. This work aims to develop a high-performance AFP-based biomaterial for cryopreservation, laying a theoretical and experimental foundation for DMSO-free cell freezing systems.

## Material and methods

### Antifreeze protein preparation

Using the pre-constructed pPIC9K/rSfAFP-His plasmid as a template [22], the recombinant plasmid pPIC9K/rSfAFP-MU was constructed via circular PCR with primers MU-F/R (plasmid map in Fig. S1A, primer details in Table S1). Site-directed mutagenesis was performed on pPIC9K/rSfAFP-MU using primers MU-D75A-F/R, MU-P81A-F/R, and MU-P78A-F/R to introduce D75A, P81A, and P78A mutations (amino acid sequences in Fig. S2A), yielding mutant plasmids pPIC9K/rSfAFP-MU-D75A, -P81A, and -P78A. For transmembrane-capable AFP, the TAT peptide (GRKKRRQRRRPPQ) was fused to the N-terminus of rSfAFP-MU using linkers including PAPAP, (G_4_S)_3_, or direct fusion, generating pPIC9K/TAT-PT-MU, -TAT-GS-MU, and -TAT-MU (synthesized by Sangon Biotech, Shanghai, China).

Recombinant plasmids were linearized with Sal I (TaKaRa, Dalian, China) at 37 °C for 3 h, then electroporated into *K. phaffii* GS115 competent cells (Invitrogen, Carlsbad, USA). The transformed cells were plated on YPD agar containing gradient concentrations of G418 (1–4 mg/mL) and incubated at 30 °C for screening of high-copy integrants. Positive clones were induced with methanol for recombinant AFP expression, followed by purification using a HisTrap™ FF column (GE Healthcare, Chicago, USA). The purified proteins were desalted, concentrated, and stored at −80 °C. For analysis, purified proteins were separated by 12 % SDS-PAGE, stained with Coomassie Brilliant Blue R250 (Thermo Fisher Scientific, Shanghai, China), and visualized using a Gel Doc system (Bio-Rad, Hercules, USA).

### TH activity assay

TH activity was evaluated using a differential scanning calorimeter (DSC8500, PerkinElmer, Shanghai, China) following a published protocol [22]. Briefly, 800 μM protein samples were sealed in aluminum sample pans and subjected to the following temperature cycle: cooling from 20 °C to −25 °C at 1 °C/min, holding for 5 min, heating to 10 °C at 1 °C/min to record ice melting temperature (T_h_), then cooling to −10 °C at 1 °C/min to determine the onset temperature of ice recrystallization (T_0_). Antifreeze activity was calculated as TH = T_h_ − T_0_, which reflects the protein's capacity to inhibit ice crystal growth.

### Protein surface hydrophobicity assay

Surface hydrophobicity was determined using 8-anilino-1-naphthalenesulfonic acid (ANS, Solarbio, Beijing, China) as a hydrophobic probe [23]. Purified AFPs were diluted to final concentrations of 50, 100, 200, and 400 μg/mL in 20 mM phosphate buffer (K_2_HPO_4_-KH_2_PO_4_, pH 7.4), followed by the addition of ANS to a final concentration of 100 μM. After thorough mixing, samples were incubated in the dark for 1 h. Fluorescence intensity was measured with a fluorometer at an excitation wavelength of 390 nm and an emission wavelength of 470 nm. A plot of fluorescence intensity versus protein concentration was generated, and the slope of the initial linear segment was defined as the surface hydrophobicity index.

### Mean particle size determination

The mean particle size of AFPs was determined using a Nanoparticle Size and Zeta Potential Analyzer (Malvern Zetasizer Nano ZS90, Malvern, UK) based on the dynamic light scattering (DLS) principle [24], to evaluate the aggregation state of recombinant AFPs. For measurements, water with a refractive index of 1.330 was used as the dispersant.

### Transmission electron microscopy (TEM) and atomic force microscopy (AFM) analysis

TEM imaging was performed using a Hitachi HT-7800 (Hitachi, Tokyo, Japan). Briefly, 10 μL of sample was dropped onto a carbon-coated grid pre-treated with air glow discharge for 1 min, followed by negative staining with 2 % phosphotungstic acid for 60 s. The grid was then observed under the Hitachi HT-7800 at an operating voltage of 80–120 kV. For AFM analysis, several drops of the dispersed solution were applied to a mica sheet, air-dried, and imaged using a Bruker Dimension Icon AFM (Bruker, Karlsruhe, Germany) with a scanning range of 1 μm × 1 μm.

### Molecular dynamics (MD) simulations

The mutant protein structures were predicted using AlphaFold3, and parallel dimer configurations with a 10 Å inter-dimer distance were constructed using PyMOL. MD simulations were performed using GROMACS 21.6 [25] with the Amber14sb force field. The simulation system was solvated in a 10 × 10 × 10 nm^3^ cubic box using the TIP3P explicit water model. Energy minimization was conducted in two stages: steepest descent (1000.0 kJ/mol/nm) followed by conjugate gradient (100.0 kJ/mol/nm). Pre-equilibration was performed under NVT and NPT ensembles for 1 ns each. Production runs of 200 ns were carried out without constraints. All bonds involving hydrogen atoms were constrained using the LINCS algorithm. Temperature (298.15 K) and pressure (1.0 bar) were controlled using the V-rescale thermostat and Parrinello-Rahman barostat with time constants of 0.1 and 2 ps, respectively. Long-range electrostatic interactions were treated using the Particle-Mesh Ewald (PME) method with a 10 Å cutoff for van der Waals interactions. A 2 fs time step was used, and snapshots were collected every 1.0 ps. Unless otherwise specified, simulation parameters followed those of previous studies [26]. The inter-monomer angle at 200 ns was visualized and measured using PyMOL, and A-B chain interactions were analyzed using Ligplot^+^ [27].

### Determination of cryoprotectant cytotoxicity

Three cell lines were used in this study: EA.hy926 (RRID: CVCL_3901), A549 (RRID: CVCL_0023), and HEK293 (RRID: CVCL_0045). All cell lines were purchased from the Cell Resource Center, Shanghai Institute for Biological Sciences, Chinese Academy of Sciences (Shanghai, China). The non-toxic concentrations of rSfAFP-series cryoprotectants were determined using a Cell Counting Kit-8 (CCK-8) assay kit (MedChemExpress, New Jersey, USA) [28]. As shown in Fig. S3, the maximum non-toxic concentrations of rSfAFP, rSfAFP-MU, rTAT-GS-MU, and Pep-1 (MedChemExpress, New Jersey, USA) in three cell lines were 800 μM, 800 μM, 400 μM, and 0.4 mg/mL, respectively. All samples in this study were used at these determined maximum non-toxic concentrations. Additionally, single-amino acid mutants of rSfAFP-MU (rMU-D75A, rMU-P81A, rMU-P78A) showed no significant cytotoxicity differences from rSfAFP-MU; thus, 800 μM was uniformly applied as the non-toxic concentration for subsequent experiments with these mutants.

### Cell culture and cryopreservation

Cells were maintained in Dulbecco’s Modified Eagle Medium (DMEM; Gibco, Grand Island, USA) supplemented with 10 % fetal bovine serum (FBS; Gibco, Grand Island, USA) and 1 % penicillin–streptomycin (Beyotime Biotechnology, Shanghai, China), and incubated at 37 °C in a humidified atmosphere containing 5 % CO_2_.

For cryopreservation, exponentially growing cells were detached with 0.25 % trypsin-EDTA (Beyotime Biotechnology, Shanghai, China), resuspended in fresh medium, counted using a Countess 3 Automated Cell Counter (Thermo Fisher Scientific, Waltham, USA), and adjusted to 1 × 10^6^ cells/mL. Cell suspensions were mixed with cryoprotectants, aliquoted into cryovials, and cooled at 1 °C /min in a Nalgene® freezing container (filled with isopropanol) from room temperature to −80 °C. After 24 h, the cryovials were transferred to liquid nitrogen vapor phase (−196 °C) with pre-chilled forceps and stored for 48 h before post-thaw viability assessment.

### The cell recovery rate and morphology analysis

Cryovials were rapidly thawed in a 37 °C water bath, stained with trypan blue (Thermo Fisher Scientific, Waltham, USA), and counted using an automated cell counter [28]. The cell recovery rate was calculated as the percentage of viable cells relative to the total number of initial cells before cryopreservation. Subsequently, cells were seeded into 6-well plates and incubated in a 37 °C, 5 % CO_2_ incubator for 24 h. Adhesion and gross morphology were examined 24 h post-thaw to obtain a rapid, qualitative impression of early survival and attachment. Morphological analysis of cryopreserved cells was performed using a Nikon Eclipse Ts2 microscope (Nikon, Tokyo, Japan).

### Fluorescent labeling

Green fluorescent labeling of rSfAFP-MU and rTAT-GS-MU was performed using the Linkine™ AbFluor™ 488 Conjugation Kit (Abbkine, Wuhan, China; Cat. No. KTL0521). The activated AbFluor™ 488 conjugate was mixed with proteins, and the conjugation reaction was carried out at 37 °C in the dark for 1 h. Fluorescently labeled proteins (rSfAFP-MU-488 and rTAT-GS-MU-488) were purified using the provided spin columns.

### Transmembrane uptake assay

The transmembrane capability of antifreeze proteins was validated using two strategies: (1) direct treatment with rTAT-GS-MU-488; (2) formation of rSfAFP-MU-488 + Pep-1 complexes by incubating rSfAFP-MU-488 with Pep-1 at a 1:1 ratio in phosphate-buffered saline (PBS; Beyotime Biotechnology, Shanghai, China) for 30 min at room temperature [19]. Cells (1 × 10^5^ cells/mL) were seeded in 15 mm glass-bottom dishes and allowed to adhere for 24 h at 37 °C in a humidified atmosphere containing 5 % CO_2_. Treatments included 100 μL of rSfAFP-MU (fluorescence control), rSfAFP-MU-488 (spontaneous uptake), rSfAFP-MU-488 + Pep-1, or rTAT-GS-MU-488. After 30 min of incubation, 100 μL serum-free DMEM was added for 1 h, followed by 100 μL complete DMEM for another hour. Cells were washed 3 times with PBS, stained with DAPI (Beyotime Biotechnology, Shanghai, China) for nuclear visualization, and imaged using a Nikon AX-SHS confocal microscope (Nikon, Tokyo, Japan). Green fluorescence (488 nm excitation) and DAPI (405 nm excitation) were visualized separately. Using ImageJ, the average fluorescence intensity of Alexa Fluor 488 in each group was quantified, with the rSfAFP-MU group set as 1 for relative comparison.

### Mitochondrial membrane potential assay

5,5′,6,6′-Tetrachloro-1,1′,3,3′-tetraethylbenzimidazolylcarbocyanine iodide (JC-1) acts as a specific fluorescent probe for mitochondrial membrane potential (ΔΨm). In healthy mitochondria, JC-1 forms J-aggregates emitting red fluorescence; upon membrane potential depolarization in apoptotic cells, it dissociates into monomers with green fluorescence. The Mitochondrial Membrane Potential Assay Kit (Elabscience Biotechnology, Wuhan, China; Cat. No: E-CK-A301) was used to assess ΔΨm in cryopreserved EA.hy926, A549, and HEK293 cells. Cells were pretreated according to the kit instructions, and fluorescence was detected using a BD FACSAria™ III flow cytometer (BD Biosciences, San Jose, USA). The intracellular mitochondrial membrane potential level was expressed as the JC-1 red/green fluorescence ratio.

### Nuclear DNA damage assay

γ-H2AX is a key marker for nuclear DNA damage and repair. Upon DNA double-strand breaks, PI3K-related kinases (ATM, ATR, etc.) phosphorylate serine 139 of the histone H2A variant H2AX, generating γ-H2AX. The DNA Damage Assay Kit (Beyotime Biotechnology, Shanghai, China; Cat. No. C2037S, containing mouse monoclonal antibody and green fluorescent label) was used to detect γ-H2AX levels in cryopreserved EA.hy926, A549, and HEK293 cells according to the manufacturer’s instructions. After staining, samples were imaged using a Nikon AX-SHS confocal microscope (Nikon, Tokyo, Japan), with γ-H2AX visualized as green fluorescence (488 nm excitation) and nuclei as blue fluorescence (405 nm excitation). Using ImageJ, the average fluorescence intensity of γ-H2AX in each group was quantified relative to the fresh cell group (set as 1).

### Statistical analysis

Each experiment was conducted independently, with a total of three replicates. The data are shown as the means ± standard deviations. The statistical analyses were performed with Origin2021 software. Differences were considered significant at p < 0.05 (*p < 0.05, **p < 0.01, and ***p < 0.001; p ≥ 0.05 was considered not significant).

## Results

### Expression and characterization of Sequence-Optimized Mutant (rSfAFP-MU)

To restore the canonical G-G-X periodicity within segment 2 of snow-flea AFP (SfAFP), we introduced three glycine substitutions (T16G/A19G/S22G) to generate rSfAFP-MU (Fig. 1C). Heterologous expression in *K. phaffii* GS115 yielded a ∼7.3 kDa protein in culture supernatants (Fig. S1B), which was purified to homogeneity via Ni-affinity chromatography for subsequent analysis.

**Fig. 2:**
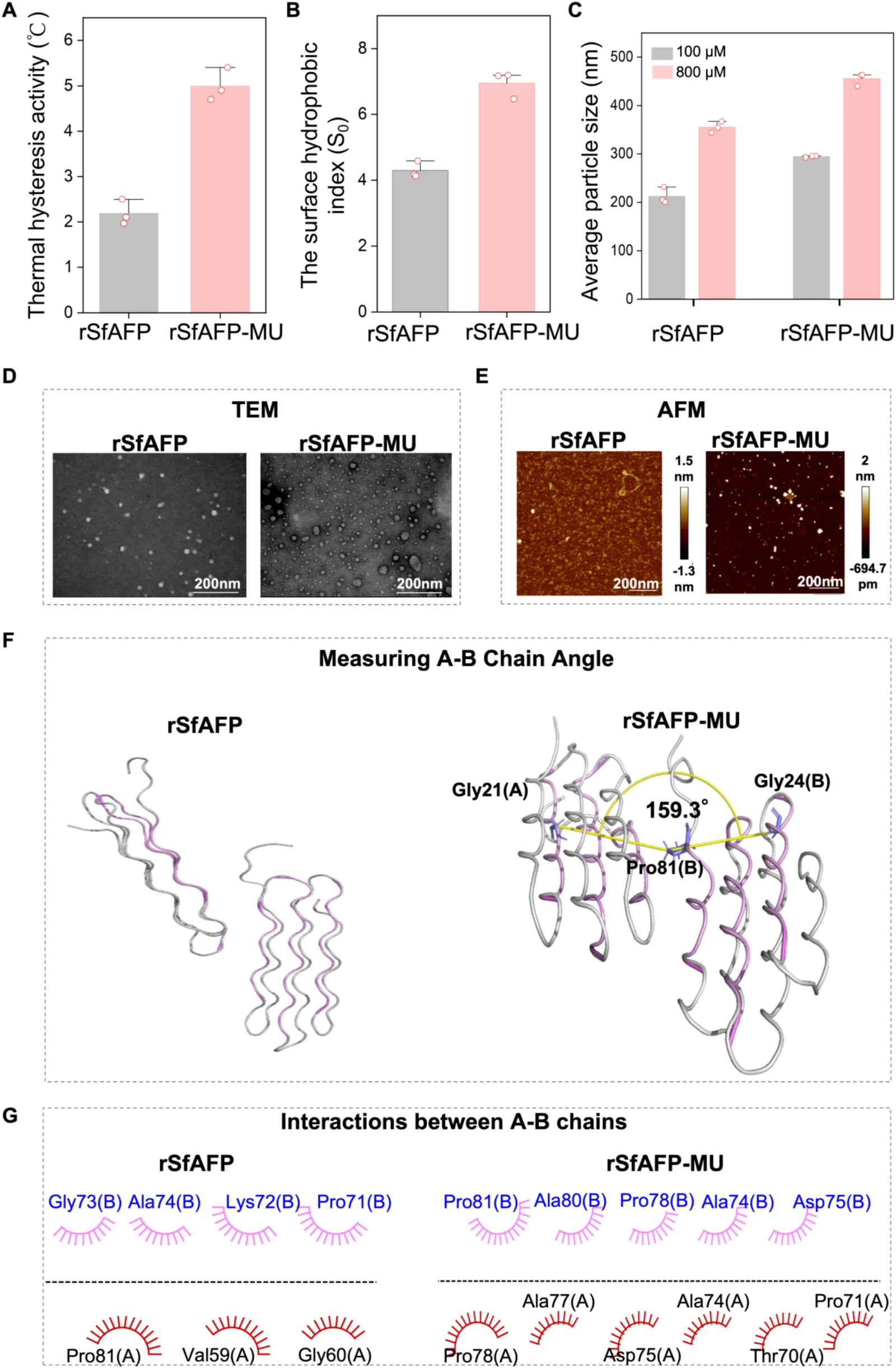
Characterization of rSfAFP-MU. (A) Thermal hysteresis activity. (B) Protein surface hydrophobicity. (C) Mean particle size distribution determined by dynamic light scattering (DLS). (D) Representative transmission electron microscopy (TEM) image. (E) Representative atomic force microscopy (AFM) image. (F) Dimer structure at 200 ns of molecular dynamics simulation: structural visualization and angle measurement were performed using PyMOL. (G) Detailed view of the dimer interaction interface generated by LigPlot^+^, highlighting key residues between chains A and B.

TH activity and surface hydrophobicity assays confirmed significant functional enhancement: relative to wild-type rSfAFP, rSfAFP-MU exhibited a 1.28-fold increase in TH activity (Fig. 2A, Table S2) and a 61 % elevation in surface hydrophobicity (Fig. 2B), with representative ANS fluorescence–concentration curves shown in figure S4A/B. Given the established link between AFP function and higher-order structures [29], we hypothesized that improved activity arose from altered self-assembly. Native PAGE revealed concentration-dependent dimerization for both proteins, with rSfAFP-MU forming dimers more efficiently at equivalent concentrations (Fig. S1C). DLS analysis further supported this finding, showing that rSfAFP-MU assembled into larger aggregates (454.47 nm average diameter at 800 μM) compared to rSfAFP (355.1 nm) (Fig. 2C); the corresponding volume-based size distributions are provided in figure S4C. TEM and AFM imaging confirmed that rSfAFP-MU formed larger, three-dimensional vesicle structures (Fig. 2D/E). Collectively, these results demonstrate that rSfAFP-MU possesses superior self-assembly capacity, which likely contributes to its enhanced antifreeze activity by providing a larger ice-binding surface area.

To further elucidate the conformational disparities between rSfAFP and rSfAFP-MU dimers, a 200-ns molecular dynamics simulation was performed (Fig. 2F). LigPlot^+^ analysis revealed that both dimers maintained structural stability through hydrophobic interactions; however, rSfAFP-MU exhibited significantly more extensive hydrophobic, with key contributing sites at Pro78, Pro81, and Asp75 (manifested as denser hydrophobic combs, Fig. 2G). Pymol visualization demonstrated that rSfAFP-MU dimers adopted a planar configuration with monomers aligned nearly parallel (159.3° angle between αC atoms of Gly21 (A) and Gly24 (B), centered on Pro81 (B) αC), forming a contiguous hydrophobic surface that effectively expanded the ice-binding interface. In contrast, rSfAFP monomers remained dispersed, failing to assemble into a cohesive ice-binding plane.

### Effects of site-directed mutations (D75A/P78A/P81A) on antifreeze performance of rSfAFP-MU

MD simulations (Fig. 2F) demonstrate that the monomeric arrangement in rSfAFP-MU dimers dictates the formation of a contiguous hydrophobic ice-binding plane, with Pro78, Pro81, and Asp75 serving as critical residues for dimer stabilization via hydrophobic interactions. These findings suggest that targeted modulation of these residues could remodel the dimer's hydrophobic core architecture and spatial orientation, thereby influencing AFP activity. To validate this hypothesis, site mutants D75A, P81A, and P78A (rMU-D75A, rMU-P81A, rMU-P78A) were designed and constructed based on rSfAFP-MU (Fig. S2A/B). Following transformation, expression, and purification, SDS-PAGE analysis (Fig. S3C) showed specific bands at 7.2 kDa for each mutant, consistent with the expected molecular weight. The purified proteins were desalted and lyophilized for subsequent functional characterization.

**Fig. 3:**
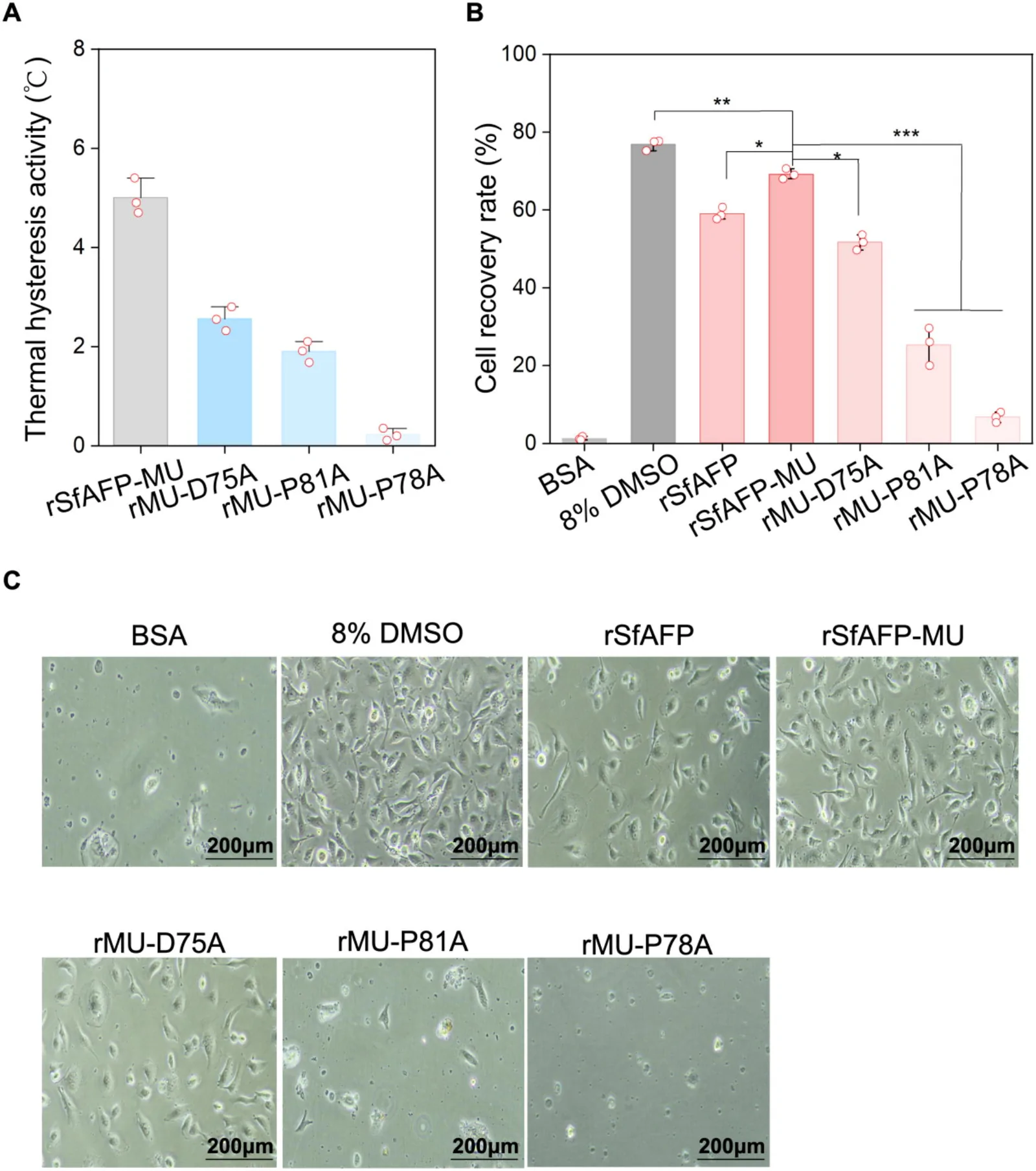
Thermal hysteresis activity analysis of rSfAFP-MU, rMU-D75A, rMU-P81A, rMU-P78A and evaluation of cryopreservation effect on EA.hy926 cells. (A) Thermal hysteresis activity. (B) Cell recovery rate after thawing detected by trypan blue staining (data expressed as the percentage of viable cells in the initial frozen cell population). (C) Morphological changes of thawed cells observed by bright-field microscopy.

Antifreeze activity assays showed that the TH activity of the three mutants (Fig. 3A, Table S2) was significantly lower than that of rSfAFP-MU, with rMU-P78A exhibiting a TH value of only 0.22°C, indicating that mutations in key amino acids directly affect the binding efficiency of the protein to ice crystals and weaken its antifreeze performance. EA.hy926 cells, which combine the biological characteristics of vascular endothelial cells with experimental controllability and reproducibility, are widely used for evaluating the efficacy of cryoprotectants [30]. Under normal conditions, EA.hy926 cells are spindle-shaped and form a continuous monolayer; under freezing stress, the cells become rounded and their junctions are disrupted [30]. Given the cryoprotective potential of rSfAFP in EA.hy926 cells, this study further investigated the cytoprotective ability of rSfAFP-MU and its mutants. Cryopreservation experiments (Fig. 3B/C) showed that cells in the bovine serum albumin (BSA) group (normal protein) almost all died, while the 8 % (v/v) DMSO group (conventional preservation conditions) had a cell recovery rate of 76.81 % and maintained a spindle-shaped network structure. The rSfAFP-MU group showed an increased cell recovery rate of 69.20 % (up from 59.00 % in the wild-type rSfAFP group) while maintaining a better spindle-shaped network structure. However, the mutant groups (rMU-D75A, rMU-P81A, rMU-P78A) exhibited significantly reduced cell recovery rates, which could be attributed to their diminished antifreeze activity. These groups were accompanied by cellular damage, including cell rounding, increased intercellular gaps, and debris formation. Notably, rMU-P78A showed the most pronounced decrease, with the cell recovery rate dropping to only 6.72 %.

### Molecular mechanism analysis of antifreeze capacity differences in rMU-D75A, rMU-P81A, and rMU-P78A

**Fig. 4:**
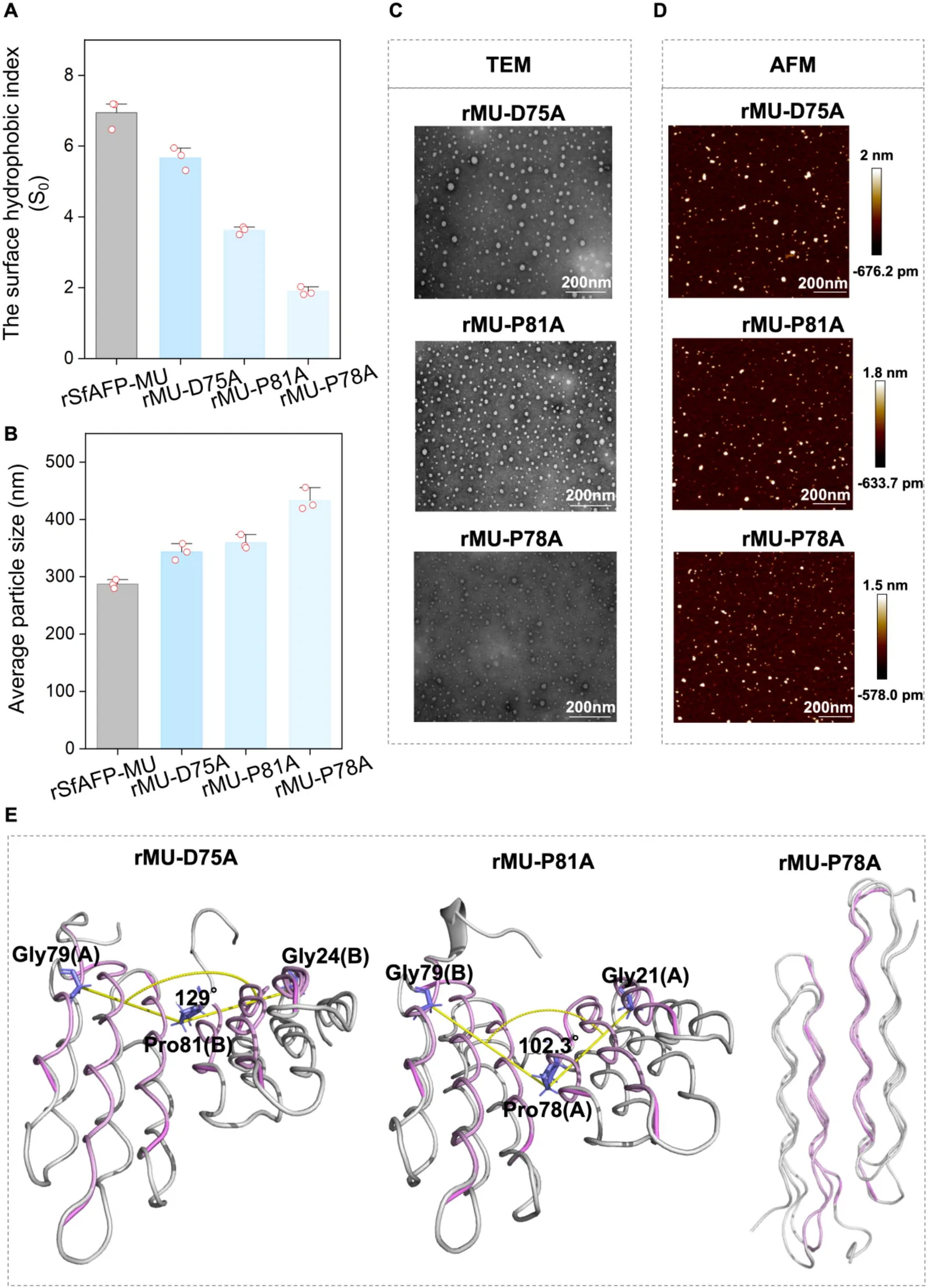
Characterization of rMU-D75A, rMU-P81A, and rMU-P78A. (A) Measurement of protein surface hydrophobicity index. (B) Determination of mean particle size by dynamic light scattering (DLS). (C) Representative transmission electron microscopy (TEM) images. (D) Representative atomic force microscopy (AFM) images. (E) Dimer structure at 200 ns from molecular dynamics simulation, with interfacial angles measured using PyMOL.

To elucidate why rMU-D75A, rMU-P81A, and rMU-P78A exhibit weaker antifreeze activity than rSfAFP-MU, we conducted the following analyses: Surface hydrophobicity index (Fig. 4A and Fig. S5A-D) correlated with TH activity, with rMU-P78A showing the lowest hydrophobicity, confirming that reduced hydrophobic regions directly impair ice-binding ability. However, DLS (Fig. 4B and Fig. S5E) showed that rMU-P78A exhibited better aggregation capacity than other mutants. TEM and AFM observations (Fig. 4C/D) further revealed that although the three mutants could form regular vesicle structures, their sizes were significantly smaller than those of rSfAFP-MU. These results indicate that antifreeze activity is not solely determined by dimerization capacity but also by the spatial conformation of dimers.

MD simulations (200 ns) of mutant dimers revealed that all three still relied primarily on hydrophobic interactions for stability, but with a significant increase in the number of amino acids involved in hydrophobic interactions (Fig. S6), consistent with DLS results, indicating enhanced hydrophobic polymerization between monomers. Specifically: In rMU-D75A, Ala75 (B) forms hydrogen bonds with Ala20 (A), strengthening dimer interface stability; In rMU-P81A, Ala81 (B) maintains structure via hydrophobic interactions with Gly25 (A), Gly3 (A), and Lys2 (A); In rMU-P78A, reduced hydrophobicity of Ala78 promotes new interactions among other amino acids. Structural analysis (Fig. 4E) showed that rMU-D75A and rMU-P81A dimers formed planar structures with angles of 129.0° and 102.3° angle, respectively, but the hydrophobic surfaces (ice-binding interfaces) had insufficient extension and lower monomer parallelism than rSfAFP-MU. The rMU-P78A dimer underwent complete hydrophobic polymerization without forming an effective ice-binding plane, leading to a significant reduction in ice-binding efficiency.

These results demonstrate that key amino acid mutations significantly reshaped the hydrophobic core structure and spatial conformation of rSfAFP-MU dimers. Among them, the steric hindrance of Pro78 is the core factor maintaining the parallel arrangement of dimers and optimizing the ice-binding interface. For SfAFP-like antifreeze proteins, the spatial morphology of dimers (especially the exposure degree of ice-binding interfaces) is more critical than dimerization ability.

### Transmembrane modification of rSfAFP-MU improves cryopreservation efficiency in EA.hy926 cells

**Fig. 5:**
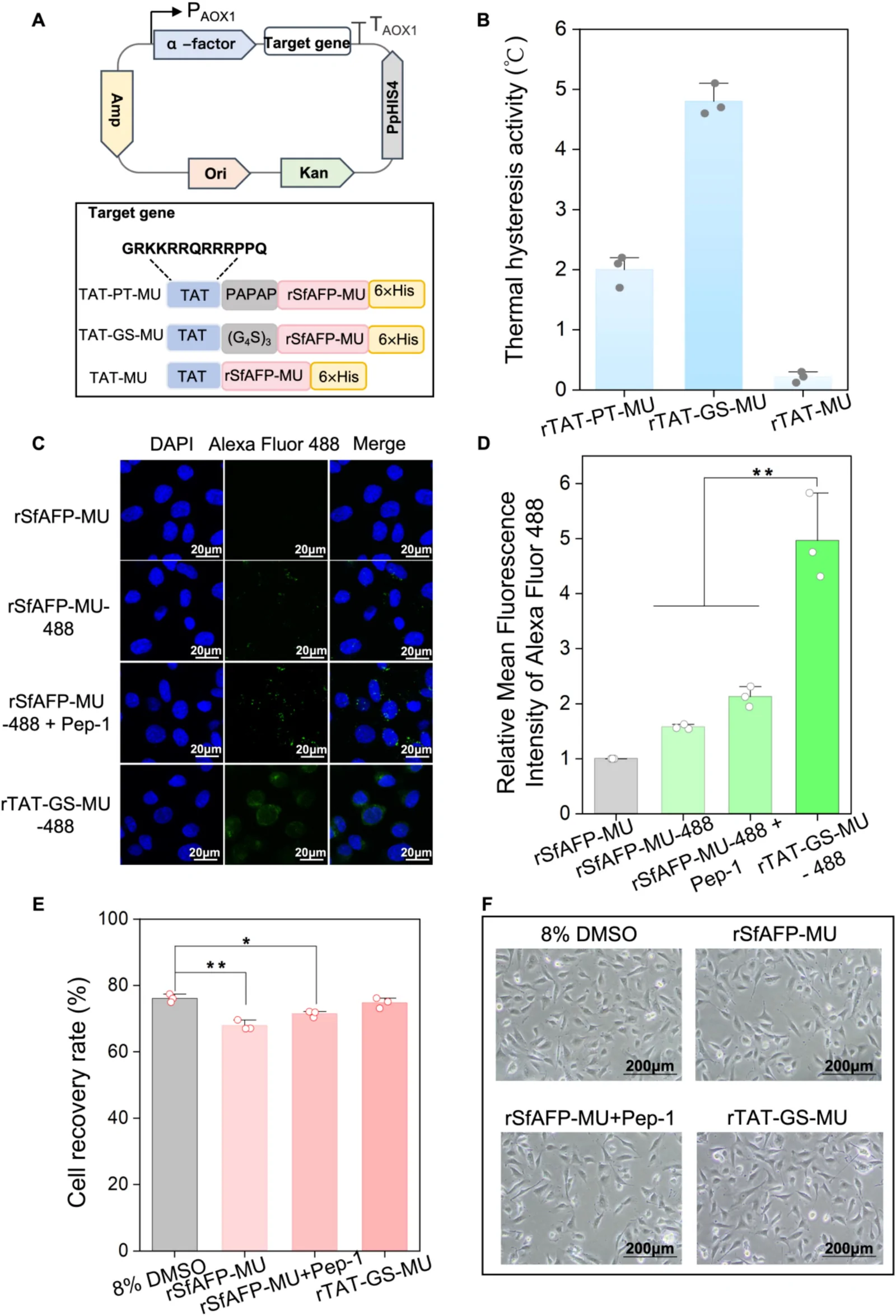
Construction and functional characterization of transmembrane AFPs (EA.hy926 cells). (A) Schematic diagram of expression vectors fusing TAT and rSfAFP-MU genes via different linkers. (B) Thermal hysteresis activity assay of fusion proteins with different linkers. (C) Confocal laser scanning microscopy for transmembrane efficiency of antifreeze proteins (green fluorescence: Alexa Fluor 488, excitation 488 nm; cell nuclei: DAPI, excitation 405 nm). (D) Quantitative analysis of mean fluorescence intensity of Alexa Fluor 488 relative to the rSfAFP-MU group (Image J software). (E) Cell recovery rate after thawing detected by trypan blue staining. (F) Morphological changes of cells after thawing observed by bright-field microscopy.

Intracellular ice formation during cryopreservation causes mechanical damage and osmotic imbalance, severely threatening cell viability and necessitating solutions for intracellular freezing damage. Most AFPs, as non-permeable protectants, inhibit extracellular ice growth but cannot address intracellular damage [5]. To overcome this limitation and enhance intracellular cryoprotective efficacy, we engineered rSfAFP-MU via two complementary strategies to endow it with transmembrane capability. First, we adopted TAT peptide fusion with rSfAFP-MU to mediate its transmembrane transport. Prior studies have shown that conjugating the TAT peptide to the N-terminus of cargo proteins preserves both cellular uptake efficiency and cargo protein activity, whereas fusing the TAT peptide to the C-terminus reduces transmembrane penetration or increases proteolytic loss [31]. Based on this finding, we fused the TAT transmembrane peptide to the N-terminus of rSfAFP-MU through three approaches [18]—PAPAP linkers, (G_4_S)_3_ linkers, or direct conjugation (Fig. 5A)—to generate recombinant plasmids pPIC9K/TAT-PT-MU, pPIC9K/TAT-GS-MU, and pPIC9K/TAT-MU. SDS-PAGE analysis confirmed the successful purification of these recombinant proteins: rTAT-PT-MU (9.4 kDa), rTAT-GS-MU (9.9 kDa), and rTAT-MU (8.9 kDa) (Fig. S7A); TH activity assays (Fig. 5B, Table S2) showed only rTAT-GS-MU retained thermal hysteresis (4.8 °C, comparable to unmodified rSfAFP-MU), leading to its selection for further validation. Such a discrepancy in TH activity among the three constructs likely originates from linker architecture. The flexible (G_4_S)_3_ spacer may act as an unstructured tether that relieves inter-domain steric clash [32], preserving the AFP ice-binding plane. In contrast, the rigid PAPAP linker could impose torsional stress on the AFP N-terminus [32], distorting its hydrophobic binding face, while direct TAT fusion might occlude the same surface through non-specific electrostatic or hydrophobic interactions of its dense cationic patch [33]. Both scenarios would plausibly attenuate TH activity. Additionally, as the second strategy, rSfAFP-MU was co-incubated with Pep-1 peptide at a 1:1 ratio to enable Pep-1-mediated intracellular translocation [11].

To quantitatively analyze intracellular protein delivery efficiency, rSfAFP-MU and rTAT-GS-MU were conjugated to AbFluor™488 for green fluorescence labeling. Conjugation results (Fig. S7B and C) showed significantly higher fluorescence intensity in SfAFP-MU-488 and rTAT-GS-MU-488 compared to unlabeled proteins (p < 0.001), confirming successful labeling. EA.hy926 cells were then incubated with rSfAFP-MU, rSfAFP-MU-488, rSfAFP-MU-488 + Pep-1, and rTAT-GS-MU-488 to compare intracellular entry efficiency. As shown in Fig. 5C and D, while rSfAFP-MU-488 partially entered cells, rSfAFP-MU-488 + Pep-1 and rTAT-GS-MU-488 exhibited significantly enhanced uptake. rTAT-GS-MU-488 showed the highest relative mean fluorescence intensity, with homogeneous green fluorescence distribution observed via confocal microscopy, whereas rSfAFP-MU-488 + Pep-1 displayed punctate patterns, indicating superior intracellular delivery efficiency of rTAT-GS-MU-488. Subsequently, the impact of transmembrane modification on post-thaw cell recovery and morphology was investigated. Results (Fig. 5E/F) revealed that compared to rSfAFP-MU, the transmembrane-capable rSfAFP-MU + Pep-1 (71.44 %) and rTAT-GS-MU (74.77 %) significantly improved the cell recovery rate. Among them, the rTAT-GS-MU group showed no significant difference from the 8 % (v/v) DMSO group (76.13 %). Cells in both groups exhibited a healthy spindle shape with tight intercellular connections.

### Effects of transmembrane − modified rSfAFP-MU on mitochondrial and nuclear DNA integrity

Mitochondrial membrane potential (ΔΨm) stability and nuclear DNA integrity are pivotal for maintaining normal cellular physiology post-thaw. During cryopreservation, both are vulnerable to disruption by osmotic fluctuations and mechanical stress [34]. While DMSO ensures a certain survival rate during freezing, its metabolic toxicity can cause severe damage to mitochondria and nuclear DNA after thawing [5]. Given that the transmembrane-modified rSfAFP-MU also exhibits favorable antifreeze activity and has shown positive effects on post-thaw cell recovery and morphology, it is critical to verify its impact on mitochondrial and nuclear DNA after thawing. Using fresh cells as controls, this study systematically compared the effects of four groups (8 % DMSO, rSfAFP-MU, rSfAFP-MU + Pep-1, rTAT-GS-MU) on ΔΨm and nuclear DNA integrity.

**Fig. 6:**
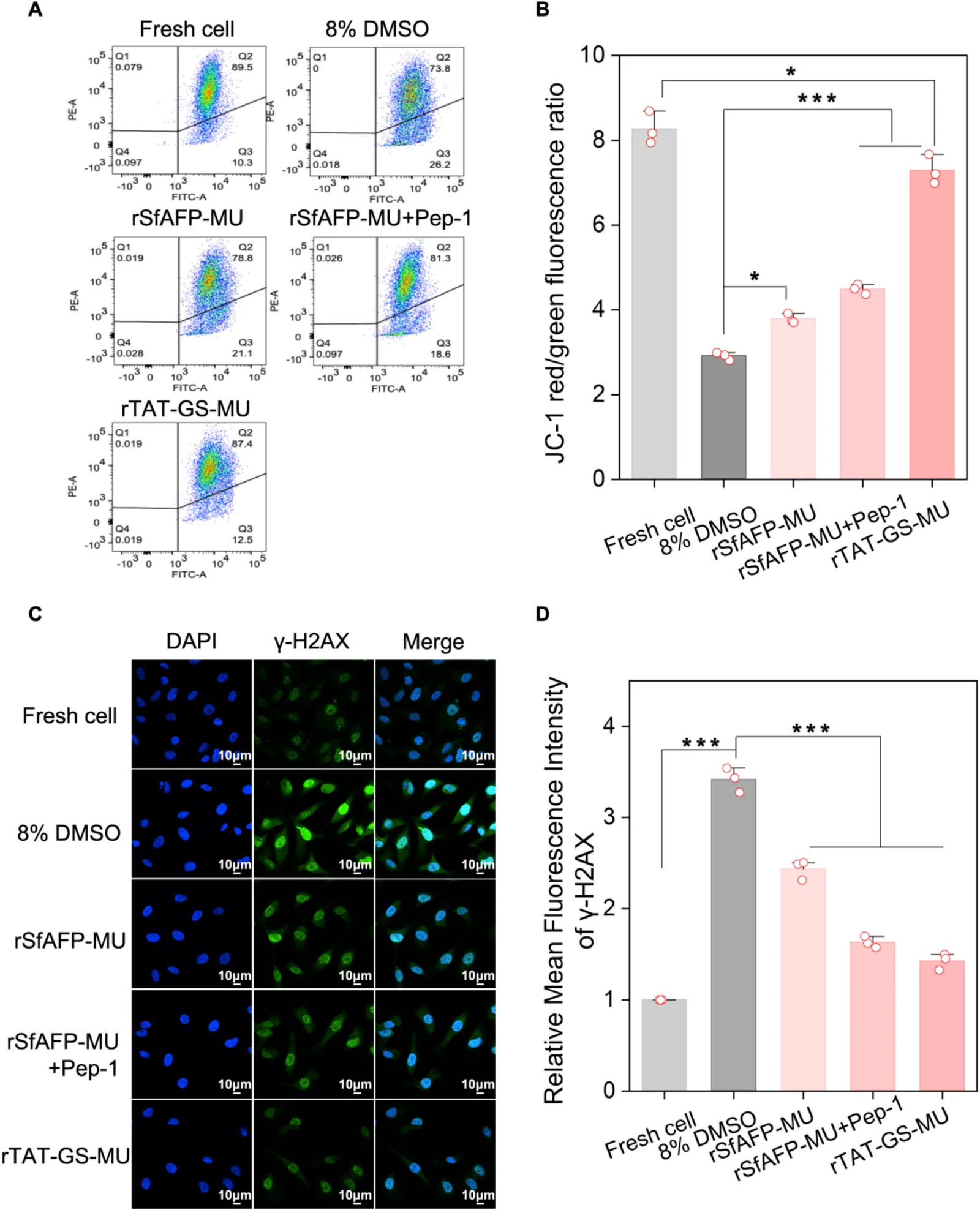
Protective effects of transmembrane AFPs on mitochondrial membrane potential and DNA damage in cryopreserved EA.hy926 cells. (A) Evaluation of mitochondrial membrane potential changes by flow cytometry in cryopreserved cells labeled with JC-1 for red/green fluorescence. The x-axis (FITC-A) corresponds to green fluorescence, and the y-axis (PE-A) corresponds to red fluorescence. Q1 (PE^+^FITC^-^) and Q2 (PE^+^FITC^+^) represent the proportion of cells with red fluorescence (normal membrane potential), while Q3 (PE^-^FITC^-^) and Q4 (PE^-^FITC^+^) represent cells with green fluorescence (decreased membrane potential). (B) Mitochondrial membrane potential levels presented as JC-1 red/green fluorescence ratios. (C) Confocal laser scanning microscopy observation of DNA damage marker γ-H2AX distribution (green fluorescence: γ-H2AX, excitation wavelength 488 nm; cell nuclei: DAPI staining, excitation wavelength 405 nm). (D) Quantitative analysis of mean γ-H2AX fluorescence intensity relative to the fresh cell group using Image J software.

JC-1, a ΔΨm-sensitive fluorescent probe, was used to assess ΔΨm via red/green fluorescence intensity ratios [35]. Flow cytometry (Fig. 6A) revealed that JC-1 aggregated in healthy mitochondria, emitting red fluorescence, whereas depolarized mitochondria in apoptotic cells induced JC-1 monomerization and green fluorescence. Results showed all groups were dominated by red fluorescence (sum of Q1 and Q2 quadrants, JC-1 aggregates): 89.58 % in fresh cells, 78.82 %/81.33 %/87.42 % in rSfAFP-MU, rSfAFP-MU + Pep-1, and rTAT-GS-MU groups, respectively, compared to 73.8 % in 8 % (v/v) DMSO. Red/green ratio analysis (Fig. 6B) demonstrated significantly higher ratios in all AFP groups versus DMSO, with rTAT-GS-MU exhibiting the greatest increase (p < 0.001) and approaching fresh cell levels. These findings confirm AFP-based cryoprotectants more effectively mitigate cryopreservation-induced mitochondrial damage than 8 % (v/v) DMSO, with rTAT-GS-MU being the most efficacious.

The γ-H2AX, a key biomarker of nuclear DNA damage[36], forms green fluorescent foci in nuclei to quantify DNA damage under different cryopreservation conditions. γ-H2AX fluorescence distribution and intensity were analyzed to assess DNA damage (Fig. 6C/D). All groups showed γ-H2AX signals with significant intensity differences: 8 % (v/v) DMSO had 3.41-fold higher relative mean fluorescence than fresh cells, confirming severe DNA damage by conventional cryoprotectants. In contrast, rSfAFP-MU, rSfAFP-MU + Pep-1, and rTAT-GS-MU groups exhibited significantly lower γ-H2AX intensity than DMSO (p < 0.001), with rTAT-GS-MU performing best. Although still differing from fresh cells, rTAT-GS-MU minimized DNA damage considering cryopreservation stress. Combined with mitochondrial membrane potential data, all AFP groups outperformed DMSO in maintaining ΔΨm and reducing DNA damage, with rTAT-GS-MU being most effective.

### Broad-spectrum cryoprotection validation of the rSfAFP-MU series

To validate the universal cryoprotective efficacy of rSfAFP-MU series proteins, this study expanded from EA.hy926 to A549 and HEK293 cells. A549 cells, with typical cancer cell biology and environmental sensitivity, are widely used in stress response and drug toxicity research [37]; HEK293 cells, normal embryonic adherent cells with high culture feasibility, transfection efficiency, and defined stress responses, serve as a standard cell biology model [38]. Representing tumor and normal cell types, these models enable comprehensive evaluation of rSfAFP-MU series across diverse cellular contexts.

To assess the universality of rSfAFP-MU series, we evaluated five metrics: membrane permeation, cell recovery, morphology, mitochondrial membrane potential, and DNA integrity. Confocal imaging revealed strongest and most uniform cytosolic fluorescence for rTAT-GS-MU-488 (Fig. S8A/S9A). All three showed green fluorescence, with rTAT-GS-MU-488 exhibiting the strongest and most uniform intracellular fluorescence. Quantification showed (Fig. S8B/S7B) its mean intracellular fluorescence was 1.26-fold (A549) and 1.69-fold (HEK293) higher than that of unmodified rSfAFP-MU-488, validating efficient delivery for downstream functional assays.

**Fig. 7:**
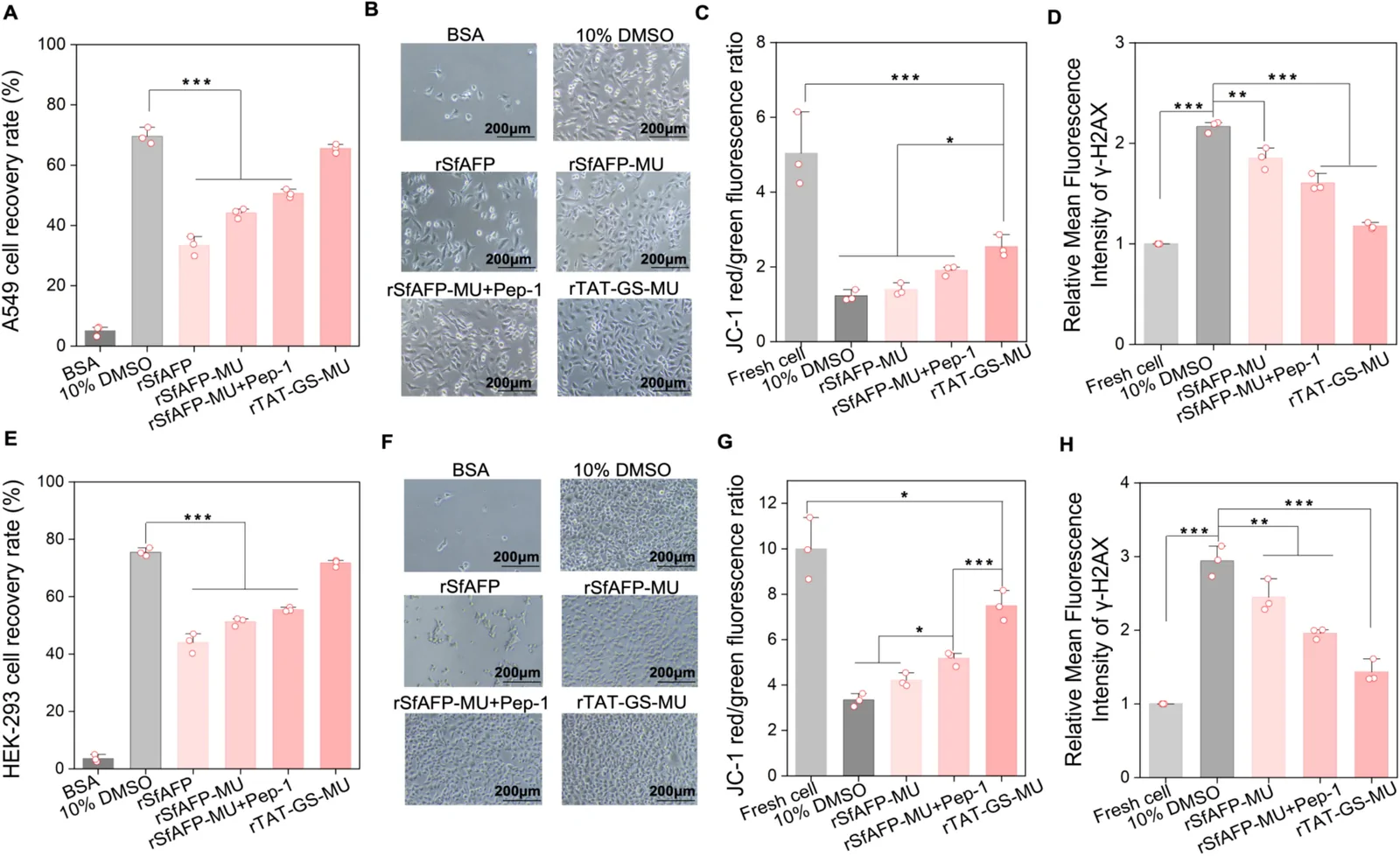
Cryoprotective effects of AFPs on A549 and HEK293 cells. (A–D) A549 cells: (A) Post-thaw viability by trypan blue exclusion assay; (B) Morphological changes via bright-field microscopy; (C) Mitochondrial membrane potential (JC-1 red/green ratio); (D) γ-H2AX foci intensity (ImageJ; normalized to fresh cells). (E–H) HEK293 cells: (E) Post-thaw viability by trypan blue exclusion assay; (F) Morphological changes via bright-field microscopy; (G) Mitochondrial membrane potential (JC-1 red/green ratio); (H) γ-H2AX foci intensity (ImageJ; normalized to fresh cells).

Next, with BSA as negative control and 10 % DMSO (routine for A549/HEK293) as positive control, AFP cryoprotective efficacy was tested. In A549 (Fig. 7A/B) and HEK293 (Fig. 7E/F) cells, BSA-treated cells were completely inactivated (round fragments). The 10 % DMSO (v/v) group showed 69.58 % (A549) and 75.45 % (HEK293) recovery; the rTAT-GS-MU group had 65.52 % (A549) and 71.70 % (HEK293), both significantly higher than other AFPs groups. For A549, both groups maintained typical squamous polygonal morphology, but rTAT-GS-MU cells were healthier with faster growth and tighter gaps. For HEK293 cells, both groups presented healthy oval morphology with similar in intercellular gaps. Among the other AFP groups, the recovery rates of the rSfAFP-MU + Pep-1 group were 50.58 % (A549)/55.47 % (HEK293), rSfAFP-MU group were 44.11 % (A549)/51.27 % (HEK293), and rSfAFP group were 33.35 % (A549)/43.97 % (HEK293); their cells had slightly widened gaps but retained basal architecture, indicating preserved cytoskeletal integrity.

Further investigation of mitochondrial membrane potential and nuclear DNA damage showed: flow cytometry in A549/HEK293 cells (Fig. S8C/S9C) revealed 10 % DMSO (v/v) group had 52.9 % and 78.05 % red fluorescent cells, significantly lower than fresh cells (86.01 %/90.8 %), confirming DMSO disrupted mitochondrial membranes. rTAT-GS-MU offered the best protection, with red fluorescent cell ratios at 69.7 % (A549) and 81.7 % (HEK293). Red/green fluorescence ratio analysis (Fig. 7C/G) indicated all AFP-treated groups outperformed DMSO, significantly improving mitochondrial membrane potential stability. Nuclear DNA damage assays showed similar trends (Fig. 7D/H, Fig. S8D/S9D): γ-H2AX fluorescence intensity in 10 % DMSO (v/v) group was 2.17-fold (A549) and 2.94-fold (HEK293) higher than fresh cells. rTAT-GS-MU reduced this intensity by 85 % (A549) and 105 % (HEK293) vs. DMSO, approaching fresh cell levels, indicating effective inhibition of cryopreservation-induced DNA double-strand breaks. In summary, SfAFP-MU series proteins had universal cytoprotection across three cell lines, with rTAT-GS-MU showing optimal performance.

## Discussion

DMSO remains the default cryoprotectant for virtually all cell banks, yet its intrinsic cytotoxicity and post-thaw metabolic disruption are now major obstacles to the clinical-scale use of cryopreserved cells [39]. Consequently, developing alternative cryoprotectants that match or surpass DMSO’s efficacy—while eliminating its toxic liabilities—has become a pressing priority. In earlier work, we expressed wild-type SfAFP in *K. phaffii*, but its modest TH rendered it impractical [22]. Here, we rationally redesigned SfAFP by (i) restoring the canonical “G-G-X” periodicity in segment 2 via site-directed mutagenesis (T16G, A19G, S22G) and (ii) fusing the resultant variant to the cell-penetrating peptide TAT to generate the engineered construct rTAT-GS-MU. Functional profiling revealed that rTAT-GS-MU exhibited a 2.68 °C increase in TH over wild-type SfAFP. When used to cryopreserve EA.hy926 endothelial cells, it achieved a 15.7 % higher post-thaw recovery than wild-type and performed on par with the standard 8 % (v/v) DMSO control. Comparable benefits were consistently observed with A549 and HEK293 cells. Notably, rTAT-GS-MU significantly outperformed DMSO in preserving mitochondrial membrane potential and reducing nuclear DNA damage, underscoring its superior long-term safety profile. These findings provide a compelling foundation for transitioning toward DMSO-free cryopreservation strategies.

SfAFP adopts a left-handed PPII helix whose amphipathic surface is compatible with Liu’s “anchoring–anti-phagocytosis” model [16]. In this framework, the IBS must present a geometrically regular array of hydrophobic side chains that complement the prism plane of ice, thereby “anchoring” the protein and arresting hexagonal ice growth propagated by hydrogen-bonded water networks [40]. This anchoring efficiency critically depends on a periodic hydrophobic platform whose spacing matches the ice lattice repeat distance. On the other hand, NBS employs strategically positioned charged or hydrophilic residues to repel nascent ice crystals via electrostatic and entropic barriers. Structural inspection of wild-type SfAFP identified Seg2 as a key IBS element; however, Seg2 deviates from the canonical “G-G-X” periodicity observed in other potent AFPs. This irregularity may disrupt the uniform spacing of hydrophobic residues, destabilizes the hydrophobic platform, and consequently attenuates ice anchoring, yielding only modest thermal hysteresis and cryoprotection [41]. To restore the missing periodicity, we introduced three site-directed mutations—T16G, A19G, and S22G—thereby reinstalling a precise “G-G-X” motif within Seg2. The resulting mutant, SfAFP-MU, exhibited a doubling of TH to 5 °C and a marked improvement in post-thaw cell recovery to 69.2 %. These gain-of-function data provide direct, quantitative support for the hypothesis that “G-G-X” periodicity is indispensable for maximal antifreeze activity in SfAFP, paralleling the essential “T-X-T” repeats documented for *Tenebrio molitor* AFP are prerequisite for ice binding [17].

The restored “G-G-X” periodicity in Seg2 appears to modulate global hydrophobicity and surface architecture. We tentatively attribute the variable dimensions of rSfAFP-MU observed by TEM/AFM to two non-mutually exclusive processes. First, both variants appear to assemble through dynamic, non-stoichiometric hydrophobic interactions that continuously nucleate and dissociate. This process yields a polydisperse mixture in which some chains form extensive oligomers while others remain only loosely or transiently associated [42]. Second, the enhanced and more patterned hydrophobic surface of rSfAFP-MU is expected to stabilize larger dimers. However, it may also introduce local kinetic bottlenecks. For instance, transient steric hindrance at aggregation-prone residues such as Pro78, or local concentration fluctuations, could plausibly arrest growth [43], occasionally yielding species smaller than the rSfAFP. Consistent with this interpretation, DLS reveals an overall increase in mean particle size (from ∼355 nm for rSfAFP to ∼454 nm), suggesting that, despite observed heterogeneity, larger aggregates are favored.

This connection between structural periodicity, aggregate characteristics, and antifreeze activity is further elucidated by molecular-dynamics simulations, which revealed the structural basis for rSfAFP-MU’s superior antifreeze efficiency. The amphipathic topology drives spontaneous dimerisation, and the relative orientation of IBS at the monomer–monomer interface is the principal determinant of ice-recognition efficiency. In this study, the rSfAFP-MU dimer adopts a 159.3° angle hydrophobic plane that maximizes geometric complementarity with the ice lattice, thereby affording a contiguous binding footprint[44]. Site-directed mutagenesis experiments showed that the P78A mutation causes topological closure of IBS, with a sharp decrease in TH activity and surface hydrophobicity index, indicating that Pro78 is the pivot maintaining this angle. In contrast, D75A and P81A mutations partially preserve the angle and binding surface through newly formed interactions, resulting in a significantly milder decrease in TH activity and hydrophobicity index. In summary, such AFPs have a hierarchical mechanism: based on the “G-G-X” periodicity to construct a regular hydrophobic platform, efficient matching with ice crystals is achieved through a specific angle of the dimer's hydrophobic surface, ultimately determining antifreeze activity.

In cell cryopreservation, DMSO’s high osmolarity displaces intracellular water but perforates membranes and generates cytotoxic by-products. In contrast, the rTAT-GS-MU protein-based protectant constructed in this study exhibits significant advantages in both “mild transmembrane transport” and “low cytotoxicity”. First, the transmembrane mode determines the difference in initial damage. DMSO relies on polar insertion into the lipid bilayer, causing irreversible changes in membrane fluidity [45]; whereas rTAT-GS-MU utilizes the electrostatic adsorption between TAT peptide and the negative charges on the membrane surface, entering cells through endocytosis, maintaining membrane integrity throughout the process, and minimizing physical damage [31]. Secondly, the protective mechanism further widens the safety gap. DMSO reduces the freezing point through osmotic dilution, which requires temporary membrane damage to complete water exchange; rTAT-GS-MU, on the other hand, relies on the specific binding between its AFP domain and the ice crystal surface to physically inhibit ice crystal growth and recrystallization, without disturbing intracellular osmotic pressure or damaging cell structure. Functional validation showed comparable EA.hy926 recovery rates (76.13 % for DMSO group vs 74.77 % for rTAT-GS-MU group) and equivalent immediate protection. However, post-thawing, DMSO induced a 1.84-fold drop in mitochondrial membrane potential and 3.41-fold higher γ-H2AX; rTAT-GS-MU remained close to fresh cells with intact organelle function, indicating superior long-term safety, validated in A549 and HEK293 cells.

To evaluate rTAT-GS-MU against existing DMSO-free CPAs, we compared it with leading candidates in this category. Synthetic polymers, including polyvinyl alcohol and poly (2-oxazoline), block ice recrystallization via hydrogen-bond networks with water molecules but remain localized extracellularly [7]. Another category of CPAs, two-dimensional materials such as GO, adsorb to ice and improve post-thawing horse sperm motility; however, they also stay extracellular, and their long-term biocompatibility remains controversial [8]. Additionally, glycoprotein-mimetic peptides exhibit marginal TH (<1 °C), which is insufficient for practical cryoprotection [9]. In contrast, rTAT-GS-MU not only retains high TH but also achieves intracellular localization via TAT-mediated delivery—directly inhibiting intracellular ice formation. Moreover, in cryopreservation experiments with EA.hy926, A549, and HEK293 cells, it shows no obvious toxicity, maintains mitochondrial and nuclear DNA integrity, and thus initially demonstrates good cytocompatibility. Overall, rTAT-GS-MU, via mild transmembrane delivery action and avoiding the accumulation of toxic by-products from DMSO metabolism, offers a safer alternative for systemic perfusion-based cell therapy, tissue engineering, and long-term organ preservation. In this proof-of-concept study, we verified that rTAT-GS-MU achieves DMSO-comparable post-thaw recovery, mitochondrial integrity, and genomic stability in target cells. Future investigations (e.g., multi-day growth curves, clonogenic assays, lineage-specific differentiation) could further extend the study’s scope to long-term proliferation assessment.

TH is traditionally used as a proxy for antifreeze protein activity, yet its direct relevance to cell survival remains contested. Several AFPs with modest TH (≤1.5 °C) confer cryoprotection comparable to high-TH variants, and synthetic polymers such as polyvinyl alcohol effectively inhibit ice recrystallization without measurable TH. These observations underscore that TH alone is insufficient to predict cryoprotective efficacy and must be interpreted alongside ice-recrystallization inhibition, nucleation suppression, and intracellular ice management. In the present work, TH was employed not as a standalone predictor, but as a quantitative engineering benchmark to validate structure-guided improvements in ice binding. The increment from wild-type rSfAFP (2.19 °C) to rSfAFP-MU (5 °C) coincided with optimized dimer geometry (159.3° hydrophobic plane) and enhanced lattice affinity, as supported by MD simulations and loss-of-function mutants (e.g., P78A, TH 0.22 °C). Functionally, this gain in TH aligned with improved post-thaw cell recovery (69 % vs 59 %), preserved mitochondrial membrane potential, and reduced γ-H2AX foci. Thus, within our AFP-centric framework, TH serves as a reliable correlate of ice-regulating capacity, while final conclusions are grounded in integrated structural, mutational, and cellular evidence.

Although this study advances AFP engineering for cryopreservation, the clinical translation of rTAT-GS-MU requires systematic evaluation. Here, we identify three bottlenecks that must be overcome to unlock its translational potential. First, regarding scalability, rTAT-GS-MU is currently produced at the laboratory scale (∼200 mg/L) in *K. phaffii* GS115 via methanol induction and Ni-affinity chromatography. Though sufficient for proof-of-concept studies, this yield is far from meeting clinical or industrial demands [46]. Second, immunogenicity is a major concern: its chimeric AFP-TAT construct has engineered hydrophobic segments (e.g., optimized Seg2) and cationic TAT residues, which may trigger innate/adaptive immune responses upon systemic exposure [47]. Third, stability limits practical use: as purified rTAT-GS-MU requires −80 °C storage, a condition incompatible with long-term distribution or clinical application. To tackle these issues, future work will explore fed-batch fermentation and high-copy integrant screening for scalability [46], and assess immunogenicity via a three-tier pipeline (*in-silico* MHC-II prediction for TAT/Seg2, *in-vitro* human peripheral blood mononuclear cells restimulation, *in-vivo* BALB/c mouse studies) [47]—with PEGylation or humanized TAT variants (e.g., R → K, Q → N substitutions) to mitigate risks if positive signals emerge [48]. Additionally, room-temperature stability will be tested under different pH, ionic strength, and excipient conditions, using trehalose or other stabilizers to extend shelf life and enable ambient storage [5].

## Conclusion

We successfully developed an engineered antifreeze protein (rTAT-GS-MU) via structural optimization and transmembrane fusion strategies, enabling efficient cryopreservation with significantly reduced cellular toxicity compared with conventional methods. By integrating molecular dynamics simulations with site-directed mutagenesis, we elucidated critical structure–activity relationships governing the ice-binding capacity of SfAFP. These advancements highlight a versatile strategy for the rational design of protein-based bioactive materials. Collectively, our findings not only advance the rational design of protein-based bioactive materials for cryopreservation applications but also establish a robust framework for developing nature-inspired alternatives to current cryoprotectants.

## Compliance with ethics requirement

Ethics approval and consent to participate: Not applicable. Experiments used only established human cell lines—EA.hy926 (RRID: CVCL_3901), A549 (RRID: CVCL_0023), and HEK293 (RRID: CVCL_0045). No human participants or animals were involved; therefore, institutional ethics approval and informed consent were not required.

## CRediT authorship contribution statement

**Yishan Fu:** Investigation, Data curation, Writing – original draft, Validation. **Yangyang Li:** Data curation. **Fei Pan:** Data curation. **Sahibzada Muhammad Aqeel:** Data curation. **Song Liu:** Conceptualization, Methodology, Supervision. **Jian Chen:** Supervision.

## Declaration of competing interest

The authors declare that they have no known competing financial interests or personal relationships that could have appeared to influence the work reported in this paper.
