# Supplementary material for: Rational engineering of an antifreeze-based biomaterial with dual-enhanced ice-binding and delivery for high-efficiency cryopreservation
Source: J Adv Res. 2025 Dec 8;87:233–47. doi: 10.1016/j.jare.2025.12.008 (PMC13539144; doi:10.1016/j.jare.2025.12.008)
Supplement: Supplementary file 1 [file mmc1.docx]

**Supplementary Material**

**Rational Engineering of an Antifreeze-based Biomaterial with Dual-Enhanced Ice-Binding and Delivery for High-Efficiency Cryopreservation**

Yishan Fu^1 ,2^, Yangyang Li^1,2^, Fei Pan^3^, Sahibzada Muhammad Aqeel^1,2^, Song Liu^1 ,2, 4*^, Jian Chen^1 ,2**^

^1^ Science Center for Future Foods, Jiangnan University, Wuxi 2141

22, China

^2^ School of Biotechnology, Jiangnan University, Wuxi 214122, China

^3^State Key Laboratory of Resource Insects, Institute of Apicultural Research, Chinese Academy of Agricultural Sciences, Beijing 100093, China

^4^JiaXing Institute of Future Food, Jiaxing, Zhejiang 314000, China

*** Corresponding authors:**

Song Liu

^*^E-mail address: liusong@jiangnan.edu.cn

Phone: +86-0510-85918309

Jian Chen

^*^E-mail address: jchen@jiangnan.edu.cn

Phone: +86-0510-85918307

**Table S1.** Primers used in this section.

| Primer name | Primer sequence (5’-3’) |
| --- | --- |
| MU-F | GTGTTAATGGTTGTCCTGGTGGTGCTGGTGGTGCTGGTGGTGTTGGTGGTC |
| MU-R | ACCAGGACAACCATTAACACCATGAGCACCATCAGCTCCCTTACACATAGCTTCAGC |
| MU-D75A-F | CTGGTACTCCAAAAGGTGCTGCTGGTGCACCTGGTGCTCCACAC |
| MU-D75A-R | AGCACCTTTTGGAGTACCAGAACCACCTTTACCACCTCTTC |
| SfAFP-His-F | GGTGCTCCACACCACCACCACCACCACTAATTCGCCTTAGACATGACTGTTCCTCAGTT |
| MU-P81A-F | GATGGTGCACCTGGTGCTGCTCACCACCACCACCACCACTAATTCGCCT |
| MU-P81A-R | AGCACCAGGTGCACCATCAGCACCTTTTGGAGTACC |
| MU-P78A-F | GTGCTGATGGTGCAGCTGGTGCTCCACACCACCAC |
| MU-P78A-R | TGCACCATCAGCACCTTTTGGAGTACCAGAACCACCTTTACC |

Note: Underlines indicate homologous arm sequences used for one-step cloning and assembly.

**Table S2.** DSC runs and calculated thermal hysteresis (TH) activities of rSfAFP and their engineered variants.

| Protein | Run | T_h_ (°C) | T_0_ (°C) | TH (°C) | Mean TH ± SD (°C) |
| --- | --- | --- | --- | --- | --- |
| rSfAFP | 1 | 2.40 | -0.10 | 2.50 | 2.19 ± 0.27 |
|  | 2 | 2.30 | 0.20 | 2.10 |  |
|  | 3 | 2.09 | 0.12 | 1.97 |  |
| rSfAFP-MU | 1 | 5.20 | -0.20 | 5.40 | 5.0 ± 0.36 |
|  | 2 | 4.20 | -0.50 | 4.70 |  |
|  | 3 | 5.01 | 0.11 | 4.90 |  |
| rMU-D75A | 1 | 2.00 | -0.80 | 2.80 | 2.56 ± 0.24 |
|  | 2 | 2.12 | -0.43 | 2.55 |  |
|  | 3 | 1.82 | -0.50 | 2.32 |  |
| rMU-P81A | 1 | 2.32 | 0.22 | 2.10 | 1.90 ± 0.21 |
|  | 2 | 2.01 | 0.10 | 1.91 |  |
|  | 3 | 1.81 | 0.13 | 1.68 |  |
| rMU-P78A | 1 | 0.10 | -0.10 | 0.20 | 0.22 ± 0.12 |
|  | 2 | -0.13 | -0.24 | 0.11 |  |
|  | 3 | 0.38 | 0.13 | 0.35 |  |
| rTAT-PT-MU | 1 | 2.30 | 0.20 | 2.10 | 2.00 ± 0.26 |
|  | 2 | 2.51 | 0.31 | 2.20 |  |
|  | 3 | 1.81 | 0.11 | 1.70 |  |
| rTAT-GS-MU | 1 | 4.58 | -0.12 | 4.70 | 4.83 ± 0.32 |
|  | 2 | 4.20 | -0.40 | 4.60 |  |
|  | 3 | 4.87 | -0.23 | 5.20 |  |
| rTAT-MU | 1 | 0.62 | 0.32 | 0.30 | 0.21 ± 0.09 |
|  | 2 | 0.39 | 0.27 | 0.12 |  |
|  | 3 | 0.12 | -0.10 | 0.22 |  |

**Figure S1.** Expression of recombinant protein SfAFP-MU. (A). Schematic diagram of the expression vector. (B). SDS-PAGE analysis: C, culture supernatant of *P.pastoris* harboring empty vector pPIC9K; 1, supernatant of *P. pastoris* expressing pPIC9K/rSfAFP-MU; 1^+^, purified rSfAFP-MU from pPIC9K/rSfAFP-MU-transformed *P. pastoris*. (C). Native PAGE analysis: 1 and 1^+^ represent 100 μM and 800 μM rSfAFP, respectively; 2 and 2^+^, 100 μM and 800 μM rSfAFP-MU, respectively. M, protein molecular weight marker. Arrows indicate the target bands.

**Figure S2.** Expression of recombinant proteins MU-D75A, MU-P81A, and MU-P78A. (A). Sequence alignment of the mutants MU-D75A, MU-P81A, and MU-P78A based on the SfAFP-MU. (B). Schematic illustration of the expression vector for the mutants. (C). SDS-PAGE analysis: C represents the culture supernatant of *P. pastoris* harboring the empty vector pPIC9K; 1, 2, and 3 denote the purified supernatants of *P. pastoris* expressing pPIC9K/MU-D75A, pPIC9K/MU-P81A, and pPIC9K/MU-P78A, respectively. M indicates the protein molecular weight marker, and arrows point to the target bands.

**Figure S3.** CCK-8 cytotoxicity assays of proteins involved in this study. (A)-(C) CCK-8 cytotoxicity results of rSfAFP, rSfAFP-MU, rTAT-GS-MU, and Pep-1 in EA.hy926 cells. (D)-(F) CCK-8 cytotoxicity results of the same proteins in A549 cells. (H)-(K) CCK-8 cytotoxicity results of the same proteins in HEK293 cells.

**Figure S4.** Surface hydrophobicity and particle-size analyses of rSfAFP and rSfAFP-MU. (A) Representative ANS fluorescence–concentration curves for rSfAFP and rSfAFP-MU (50–400 µg/mL); slopes yield surface-hydrophobicity indices. (B) DLS particle-size distributions of rSfAFP and rSfAFP-MU. Data are mean ± SD (n = 3).

**Figure S5.** Surface hydrophobicity and particle-size analyses of rSfAFP-MU and its mutants. (A)–(D) Representative ANS fluorescence–concentration curves (50–400 µg/mL) for rSfAFP-MU, rMU-D75A, rMU-P81A and rMU-P78A; slopes yield surface-hydrophobicity indices. (E) DLS particle-size distributions of rSfAFP-MU and its mutants. Data are mean ± SD (n = 3).

**Figure S6.** Intermonomeric interactions in the dimeric structures of recombinant proteins MU-D75A, MU-P81A, and MU-P78A visualized by LigPlot^+^ for A-B chain interactions. Panels (A), (B), and (C) correspond to MU-D75A, MU-P81A, and MU-P78A, respectively.

**Figure S7.** Expression of Transmembrane Proteins (rTAT-PT-MU/ rTAT-GS-MU/ rTAT-MU) and Fluorescent Labeling of rSfAFP-MU/ rTAT-GS-MU. (A). SDS-PAGE analysis of rTAT-PT-MU/ rTAT-GS-MU/ rTAT-MU: C denotes the culture supernatant of *P.pastoris* harboring empty vector pPIC9K; 1, 2, and 3 represent the purified supernatants of *P. pastoris* expressing pPIC9K/TAT-PT-MU, pPIC9K/TAT-GS-MU, and pPIC9K/TAT-MU, respectively. M is the protein molecular weight marker, and arrows indicate the target bands. (B) and (C) show the 488-specific fluorescence intensity (AU) of rSfAFP-MU and rTAT-GS-MU before and after green fluorescent labeling, respectively.

**Figure S8.** Transmembrane efficiency of transmembrane antifreeze proteins in A549 cells and their protective effects on mitochondrial membrane potential and DNA damage after cryopreservation. (A) Confocal laser scanning microscopy images of antifreeze protein transmembrane efficiency (green fluorescence: Alexa Fluor 488, excitation at 488 nm; cell nuclei: DAPI, excitation at 405 nm). (B) Quantitative analysis of mean Alexa Fluor 488 fluorescence intensity relative to the rSfAFP-MU group, performed using ImageJ software. (C) Assessment of mitochondrial membrane potential changes by flow cytometry after JC-1 labeling (red/green fluorescence) in cryopreserved cells. (D) Confocal laser scanning microscopy visualization of DNA damage marker γ-H2AX distribution (green fluorescence: γ-H2AX, excitation at 488 nm; cell nuclei: DAPI staining, excitation at 405 nm).

**Figure S9.** Transmembrane efficiency of transmembrane antifreeze proteins in HEK293 cells and their protective effects on mitochondrial membrane potential and DNA damage after cryopreservation. (A) Confocal laser scanning microscopy images of antifreeze protein transmembrane efficiency (green fluorescence: Alexa Fluor 488, excitation at 488 nm; cell nuclei: DAPI, excitation at 405 nm). (B) Quantitative analysis of mean Alexa Fluor 488 fluorescence intensity relative to the rSfAFP-MU group, performed using ImageJ software. (C) Assessment of mitochondrial membrane potential changes by flow cytometry after JC-1 labeling (red/green fluorescence) in cryopreserved cells. (D) Confocal laser scanning microscopy visualization of DNA damage marker γ-H2AX distribution (green fluorescence: γ-H2AX, excitation at 488 nm; cell nuclei: DAPI staining, excitation at 405 nm).
